# Mitochondrial metabolism-related signature depicts immunophenotype and predicts therapeutic response in testicular germ cell tumors

**DOI:** 10.1097/MD.0000000000035120

**Published:** 2023-09-15

**Authors:** Dandan Qiu, Lingling Gao, Shuo Zhang, Gang Lin, Xingwei Yu

**Affiliations:** a Department of Urology, The First Affiliated Hospital of Zhejiang Chinese Medical University, Hangzhou, Zhejiang, China; b Department of Urology, The First Affiliated Hospital, Zhejiang University School of Medicine, Hangzhou, Zhejiang, China; c Department of Breast Surgery, The First Affiliated Hospital of Zhejiang Chinese Medical University, Hangzhou, Zhejiang, China; d Department of Radiotherapy, The First Affiliated Hospital of Zhejiang Chinese Medical University, Hangzhou, Zhejiang, China.

**Keywords:** consensus clustering, immune infiltration, mitochondria, nomogram, testicular germ cell tumor

## Abstract

In recent years, there has been growing evidence linking mitochondrial dysfunction to the development and progression of cancer. However, the role of mitochondrial metabolism-related genes (MMRGs) in testicular germ cell tumor (TGCT) remains unclear. We downloaded clinical pathology, transcriptome, and somatic mutation data for TGCT from public databases and conducted univariate Cox regression analysis to investigate prognostic correlations. We also used consensus clustering to identify molecular subtypes, comparing differential expression genes, biological processes, Kyoto Encyclopedia of Genes and Genomes pathways, mutations, prognosis, immune infiltration, drug sensitivity, and immune therapeutic response between these subtypes. We constructed multi-gene risk features and nomograms for TGCT prognosis. Fifteen MMRGs were significantly correlated with progression-free survival in TGCT patients. Based on these genes, we identified 2 molecular subtypes which showed significant differences in somatic mutations, prognosis, and immune cell infiltration. These subtypes could also indicate drug sensitivity and immune therapeutic response; the subtype with poor prognosis showed a higher potential benefit from some drugs and immunotherapy. Abnormalities in immune-related biological processes and extracellular matrix as well as Kyoto Encyclopedia of Genes and Genomes pathways such as PI3K-AKT signaling pathway, pat5hways in cancer, primary immunodeficiency, and neutrophil extracellular trap formation were associated with significant differences in phenotypes among subtypes. Finally, we constructed an 8-gene TGCT risk feature based on differential expression genes between subtypes which performed well in TGCT patient prognostic evaluation. Our study elucidated the prognostic correlation between MMRGs and TGCT and established MMRG-derived molecular subtypes and risk features for personalized treatment of TGCT which have potential clinical application value.

## 1. Introduction

Testicular germ cell tumor (TGCT) is a prevalent solid tumor that occurs in young men aged 20 to 40 years old and is one of the leading causes of solid tumor death in this age group.^[1]^ The 2 major categories of TGCT are seminoma and non-seminoma, with seminoma being the more common among germ cell tumors.^[2]^ The incidence rate of TGCT is currently between 0.002% to 0.005%, and it is increasing worldwide.^[1,3]^ Unfortunately, 15% to 30% of patients with TGCT experience relapse and metastasis, which often leads to poor prognosis.^[4]^ Although conventional surgical resection and chemotherapy can cure TGCT with a cure rate over 90%, approximately 15% of TGCT patients are resistant to chemotherapy, which raises concerns about their survival status.^[5–7]^ Therefore, it is crucial to understand subtype-specific molecular mechanisms and develop sensitive biomarkers for better treatment strategies.

Mitochondria are essential organelles that play a critical role in various cellular activities such as energy metabolism, signal transduction, cell growth, and death.^[8]^ Mitochondrial dysfunction has significant impacts on cellular biological functions that lead to disease development,^[9]^ including carcinogenesis.^[10,11]^ Genomic analysis reveals that nuclear-mitochondrial genomic alterations associated with mitochondrial function are related to 38 types of tumors.^[12]^ Currently, the mitochondrial pathway has been extensively studied as a potential cancer treatment target.^[13]^ For instance, aloe vera gel polysaccharides induce colon cancer cell death through PINK1/Parkin-mediated mitophagy induced by mitochondrial damage,^[14]^ while Wang et al^[15]^ developed a new imidazo [1,2-a] pyridine derivative that exerts anticancer activity by inducing cell apoptosis through the mitochondrial pathway. Studies have reported that defects in mitochondrial-related genes can regulate the tumor immune metabolic microenvironment.^[16]^ However, the prognostic and immune value of mitochondrial metabolism-related genes (MMRGs) in TGCT has not been reported.

Therefore, this study aims to explore the potential of MMRGs as biomarkers for TGCT prognosis and treatment response by integrating TGCT transcriptome and clinical data from gene expression omnibus (GEO) and the cancer genome atlas (TCGA) databases while considering a comprehensive analysis of previous research.

## 2. Materials and methods

### 2.1. Data collection and preprocessing

The training set comprised TGCT patient expression data, somatic mutation data, and clinical information data sourced from the official website of the cancer genome atlas (TCGA: http://cancergenome.nih.gov/). Patients with incomplete prognostic information were excluded, resulting in a total of 128 patients eligible for analysis. The validation set was obtained from the GEO database (https://www.ncbi.nlm.nih.gov/geo/) and included the GSE3218 and GSE10783 datasets. To eliminate batch effects, we integrated these datasets using the “SVA” R package. Finally, we included 108 TGCT patients in the validation dataset. We acquired a total of 1234 MMRGs from previous studies through the molecular signatures database website (http://www.broadinstitute.org/gsea/msigdb/index.jsp).^[17]^

### 2.2. Consensus clustering

Univariate Cox regression analysis was employed to identify MMRGs significantly associated with TGCT prognosis. Based on this analysis, molecular subtypes of TCGA-TGCT cohort were established. Unsupervised consensus clustering was performed using the “Consensus Cluster Plus” R package based on k-means machine learning algorithm to partition or compress cases into multiple different clusters according to provided markers or signatures. The consensus clustering algorithm was iterated 1000 times with 80% of data sampled in each iteration. The optimal number of clusters was determined by Item-Consensus plot and relative changes under cumulative distribution function curve.

### 2.3. Immune infiltration analysis

We used the CIBERSORT R package to analyze immune cell infiltration status among 22 types of immune cells and applied this package to perform tumor immune cell infiltration analysis on the TCGA-TGCT cohort.

### 2.4. Immune therapy response analysis

The tumor immune dysfunction and exclusion (TIDE) algorithm is a computational model that predicts the response of tumor cells to immune therapy. This prediction is based on the expression profile of tumor cells as well as the degree of immune cell infiltration. To evaluate immune therapy response in TGCT patients, normalized transcriptome data from the TCGA-TGCT cohort was submitted to the TIDE website (http://tide.dfci.harvard.edu/) for calculation. The resulting scores, including TIDE, cancer-associated fibroblasts (CAF), Merck18, and exclusion were used for this purpose.

### 2.5. Drug sensitivity analysis

A drug sensitivity analysis was performed on patients from the TCGA-TGCT cohort using the pRRophetic R package. Six commonly used chemotherapy drugs including docetaxel, paclitaxel, vinblastine, cisplatin, doxorubicin, and etoposide were selected for this analysis.

### 2.6. Gene set enrichment analysis

The ClusterProfiler R package was utilized to perform Gene Ontology biological process and Kyoto Encyclopedia of Genes and Genomes pathway enrichment analysis on molecular subtypes derived from MMRGs. The top 5 significantly inhibited and activated biological processes and Kyoto Encyclopedia of Genes and Genomes pathways were screened for visualization.

### 2.7. Mutation analysis

The Maftools R package was employed for gene mutation analysis to calculate tumor mutation burden for each patient in the TCGA-TGCT cohort. Gene mutation waterfall plots were generated to compare gene mutations among different molecular subtypes.

### 2.8. Construction of prognostic risk signature

Firstly, differential gene expression analysis between molecular subtypes derived from MMRGs was performed using limma R package to screen genes with *P* < .01 for univariate Cox regression analysis to identify prognostic-related genes. Lasso Cox regression analysis was conducted using glmnet R package to reduce prognostic factors followed by multivariate Cox regression analysis to identify independent prognostic genes. The risk feature was calculated using the following formula: risk score =. (“i”=the number of prognostic genes, “βi” represents the expression of each gene, “coefi” represents the coefficient of each gene). Patients were divided into high-risk and low-risk groups using median values and survival analysis was performed.

### 2.9. Nomogram construction and evaluation

To assess the prognostic relevance of clinical-pathological features and risk scores derived from MMRGs in the TCGA-TGCT cohort, univariate and multivariate Cox regression analyses were conducted. The nomogram was constructed and visualized using the rms R package, while its predictive performance was evaluated through calibration curves and receiver operating characteristic curves.

### 2.10. Statistical analysis

All data analysis and visualization were performed using R software (version 4.2.2, R Foundation for Statistical Computing, Vienna, Austria). Comparisons between 2 groups were conducted using the Wilcoxon test, while survival analysis between subtypes was carried out using Kaplan–Meier plots and log-rank tests. A statistically significant difference was defined as *P* < .05.

## 3. Results

### 3.1. Prognostic relevance of MMRGs in TGCT

**Figure 1.**
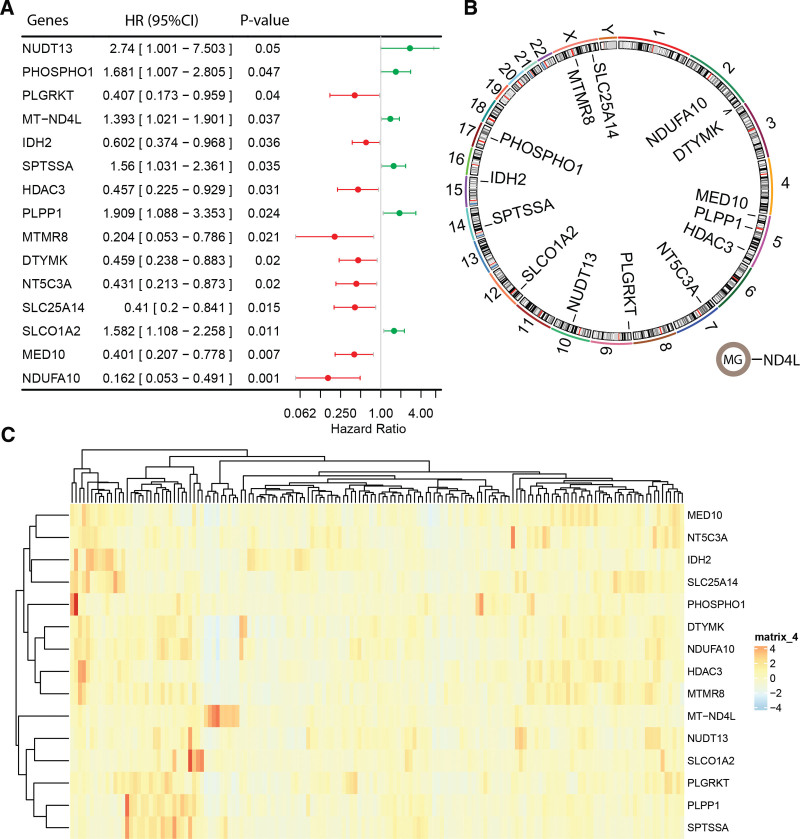
Identification of Prognostic Relevant Mismatch Repair Genes Associated with Testicular Germ Cell Tumor Prognosis: (A) forest plot displaying univariate Cox regression analysis results identifying prognostic relevant MMRGs, (B) chromosomal locations of prognostic relevant MMRGs, and (C) Expression heatmap of prognostic relevant MMRGs in the TCGA-TGCT cohort. MG = mitochondrial genome, MMRGs = mitochondrial metabolism-related genes, TCGA = the cancer genome atlas, TGCT = testicular germ cell tumor.

The prognostic relevance of MMRGs in TGCT was evaluated in the TCGA-TGCT cohort. Detailed clinical-pathological information of the TCGA-TGCT cohort, consisting of 128 patients with complete prognostic information, is presented in Table 1. Among them, 39 (30.47%) had disease progression as their outcome and 68 (53.13%) had seminoma as their histological type. Univariate Cox regression analysis identified 15 MMRGs associated with progression-free survival (PFS) in TGCT out of a total of 1234 MMRGs (Fig. 1A). Of these, 6 genes (nudix hydrolase 1, phosphatase, orphan 1, ND4L, Serine palmitoyltransferase small subunit A, PLPP1, SLCO1A2) had HR > 1 indicating an increased risk of disease progression or death in TGCT patients; while 9 genes (PLGRKT, IDH2, histone deacetylase 3 [HDAC3], MTMR8, deoxythymidylate kinase, NT5C3A, SLC25A14, mediator complex subunit 10, NDUFA10) had HR < 1 indicating a decreased risk of disease progression or death in TGCT patients. Figure 1B shows the chromosomal locations of these genes with ND4L located within the mitochondrial genome. The expression heatmap of these prognostic relevant MMRGs in the TCGA-TGCT cohort is shown in Figure 1C.

**Table 1:** The clinicopathological characteristics of the included patients in the TCGA-TGCT cohort.

Characteristics	TCGA-TGCT cohort (n = 128)
Age, yr, median (IQR)	31 (26, 67)
Status	
Progression	39 (30.47%)
Non-progression	89 (69.53%)
Laterality	
Left	68 (53.13%)
Right	55 (42.97%)
Both	5 (3.91%)
Serum_markers	
S0	42 (32.81%)
S1	36 (28.13%)
S2	33 (25.78%)
S3	4 (3.13%)
SX	12 (9.38%)
Unknown	1 (0.78%)
Pathology histology	
Non-Seminoma	60 (46.88%)
Seminoma	68 (53.13%)
Stage	
I	55 (42.97%)
II	17 (13.28%)
II	14 (10.9%)
IS	41 (32.03%)
Unknown	1 (0.78%)
T stage	
T1	70 (58.59%)
T2	53 (41.41%)
T3	5 (3.91%)
N stage	
N0	75 (58.59%)
N1	23 (17.97%)
N2	3 (2.34%)
N3	3 (2.34%)
NX	24 (18.75%)
M stage	
M0	121 (94.53%)
M1	7 (5.47%)
Radiotherapy	
Yes	24 (18.75%)
No	104 (81.25%)
Drug_therapy	
Yes	61 (47.66%)
No	67 (52.34%)
Follow-up, d, median (IQR)	1364 (694, 7437)

TCGA = the cancer genome atlas, TGCT = testicular germ cell tumor.

### 3.2. Molecular subtyping of TGCT based on MMRG

**Figure 2.**
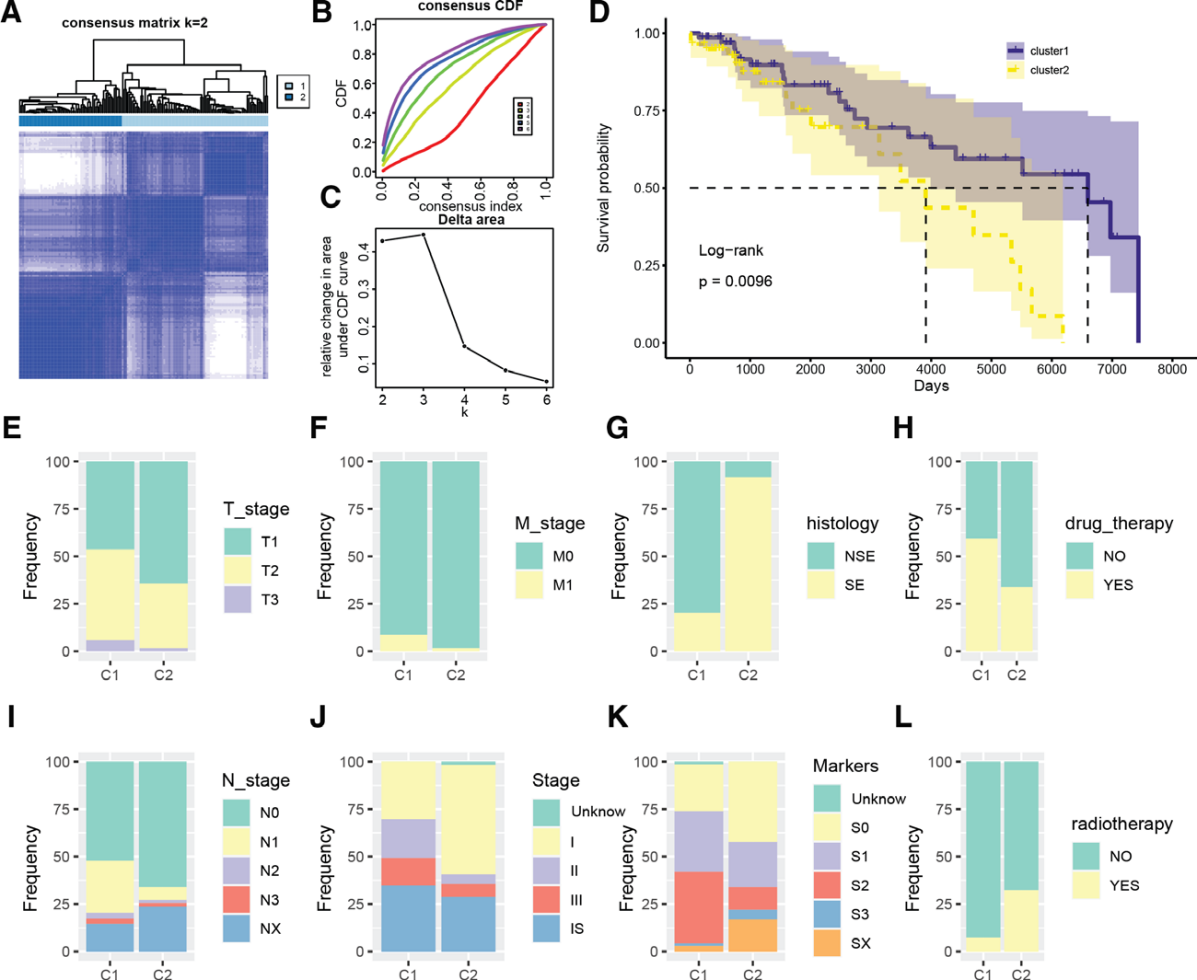
TGCT molecular subtyping based on 25 prognostic-related MMRGs. (A–C) Consensus clustering based on 25 prognostic-related MMRGs. (D) Kaplan–Meier survival curves for molecular subtypes derived from MMRGs. (E–L) Composition differences in T stage, M stage, histological type, drug treatment, N stage, clinical stage, serum markers, and radiotherapy between molecular subtypes derived from MMRGs. C1: cluster1; C2: cluster2. MMRGs = mitochondrial metabolism-related genes, TGCT = testicular germ cell tumor.

In this study, we conducted consensus clustering analysis on the TCGA-TGCT cohort utilizing 15 prognostic-related MMRGs. The results revealed 2 distinct molecular subtypes, namely cluster1 and cluster2, as illustrated in Figure 2A–C. Survival analysis demonstrated that cluster1 had a significantly longer progression-free survival than cluster2 (log-rank test: *P* = .0096), as shown in Figure 2D. To further investigate the association between MMRG-derived molecular subtypes and clinical-pathological features, we compared the distribution of clinical-pathological characteristics between different subtypes, as presented in Figure 2E–L. Our findings indicated that subtype C2 had a higher proportion of T1, M0, N0, Stage I, and S0 serum markers compared to subtype C1. Moreover, most cases of subtype C2 were seminomas. Furthermore, the proportion of patients receiving drug treatment was higher in subtype C2 while the proportion receiving radiotherapy was lower.

### 3.3. Differences in immune landscape and immunotherapeutic response between MMRG-derived molecular subtypes

**Figure 3.**
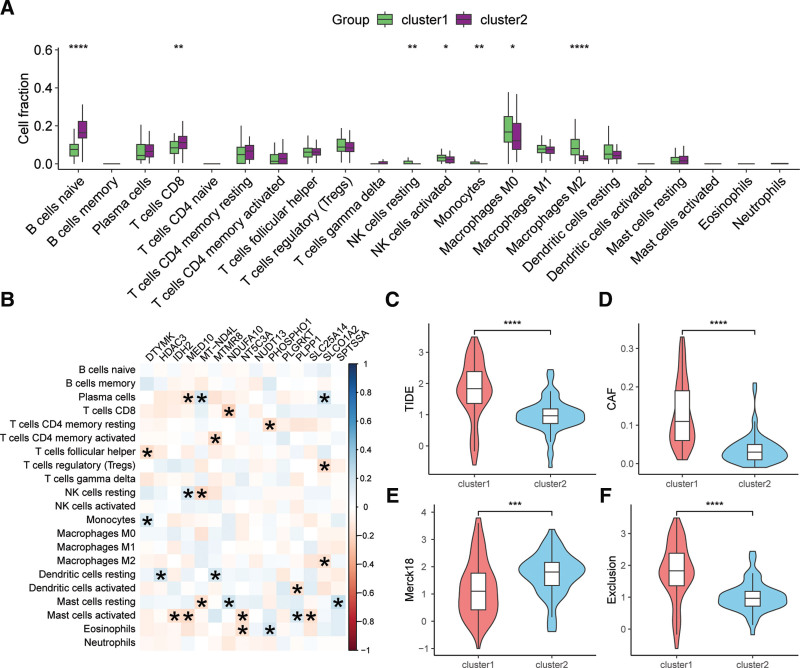
Analysis of tumor-infiltrating immune cells and immunotherapeutic response between molecular subtypes derived from MMRG in TGCT. (A) Comparison of infiltrating 22 types of immune cells between cluster1 and cluster2 subtypes. (B) Correlation analysis between 25 prognostic-related MMRGs and immune cell infiltration. (C–F) Analysis and comparison of immunotherapeutic response based on TIDE, CAF, Merck18, and exclusion scores. * *P* < .05, ** *P* < .01, *** *P* < .001, **** *P* < .0001. CAF = cancer-associated fibroblasts. MMRGs = mitochondrial metabolism-related genes, TGCT = testicular germ cell tumor, TIDE = tumor immune dysfunction and exclusion.

As depicted in Figure 3A, the TCGA-TGCA cohort exhibited a diverse array of tumor-infiltrating immune cells, including naive B cells and CD8 T cells. In contrast to cluster1, cluster2 subtype displayed significantly elevated levels of infiltrating naive B cells and CD8 T cells, while showing a significant decrease in NK cells, monocytes, M0 and M2 macrophages. Correlation analysis demonstrated that gene expression, except for PLGRKT, was significantly associated with immune cell infiltration (Fig. 3B), implying that these genes may have a potential role in shaping the tumor immune microenvironment. Further evaluation of the immunotherapeutic response of cluster1 and cluster2 subtypes revealed that compared to cluster1, cluster2 subtype had lower TIDE, CAF, and exclusion scores but higher Merck18 score, indicating an improved immunotherapeutic response (Fig. 3C–F).

### 3.4. Prediction of drug sensitivity among MMRG-derived molecular subtypes

**Figure 4.**
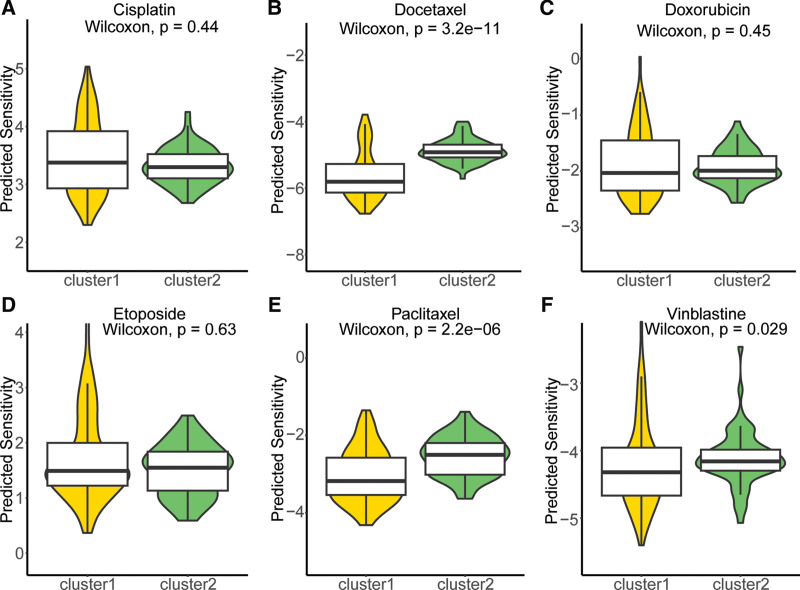
Analysis of drug sensitivity among molecular subtypes derived from MMRG in TGCT. (A–F) Comparison of sensitivity to cisplatin, docetaxel, doxorubicin, etoposide, paclitaxel, and vinblastine between cluster 1 and cluster 2 subtypes. TGCT = testicular germ cell tumor.

To further investigate the differential drug sensitivity among molecular subtypes derived from MMRG, we compared the sensitivity of cluster 1 and cluster 2 subtypes to 6 commonly used chemotherapy drugs. The findings revealed (Fig. 4A–F) that compared to cluster 2, cluster 1 subtype exhibited higher sensitivity towards docetaxel, paclitaxel, and vinblastine, while no significant difference in sensitivity was observed for cisplatin, doxorubicin, and etoposide.

### 3.5. Biological processes, pathways, and mutation characteristics among MMRG-derived molecular subtypes

**Figure 5.**
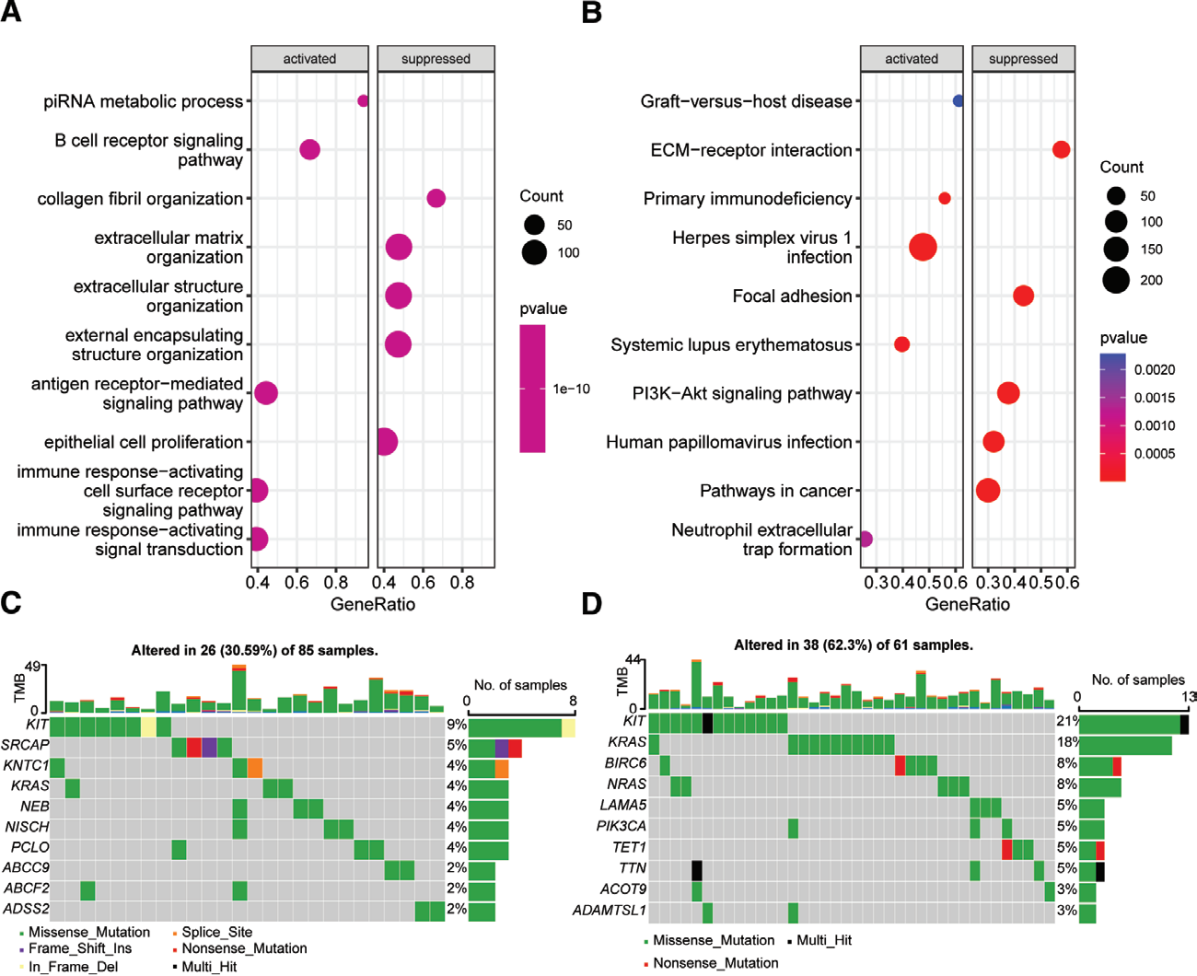
Enrichment analysis of biological processes and KEGG pathways among molecular subtypes derived from MMRG in TGCTs. (A) Biological processes that were significantly activated or suppressed in cluster2 compared to cluster1. (B) KEGG pathways that were significantly activated or suppressed in cluster2 compared to cluster1. Waterfall plots show the top 10 driver genes with the highest mutation frequency in cluster1 (C) and cluster2 (D). KEGG = Kyoto Encyclopedia of Genes and Genomes, TGCT = testicular germ cell tumor.

Gene set enrichment analysis of the MMRG-derived molecular subtypes in TGCTs revealed that immune-related biological processes, including antigen receptor-mediated signaling pathway, immune response-activating cell surface receptor signaling pathway/transduction, and B-cell receptor signaling pathway, were significantly activated in cluster 1 compared to cluster 2. Conversely, extracellular matrix-related biological processes were significantly suppressed (Fig. 5A). Furthermore, the PI3K-AKT signaling pathway and pathways in cancer were significantly suppressed while primary immunodeficiency and neutrophil extracellular trap formation were significantly activated in cluster 1 (Fig. 5B). The KIT gene was found to be the most commonly mutated gene in TGCTs across both clusters. However, there were significant differences in other gene mutation patterns between cluster 1 and cluster 2 (Fig. 5C and D).

### 3.6. Development of prognostic risk features of TGCT

**Figure 6.**
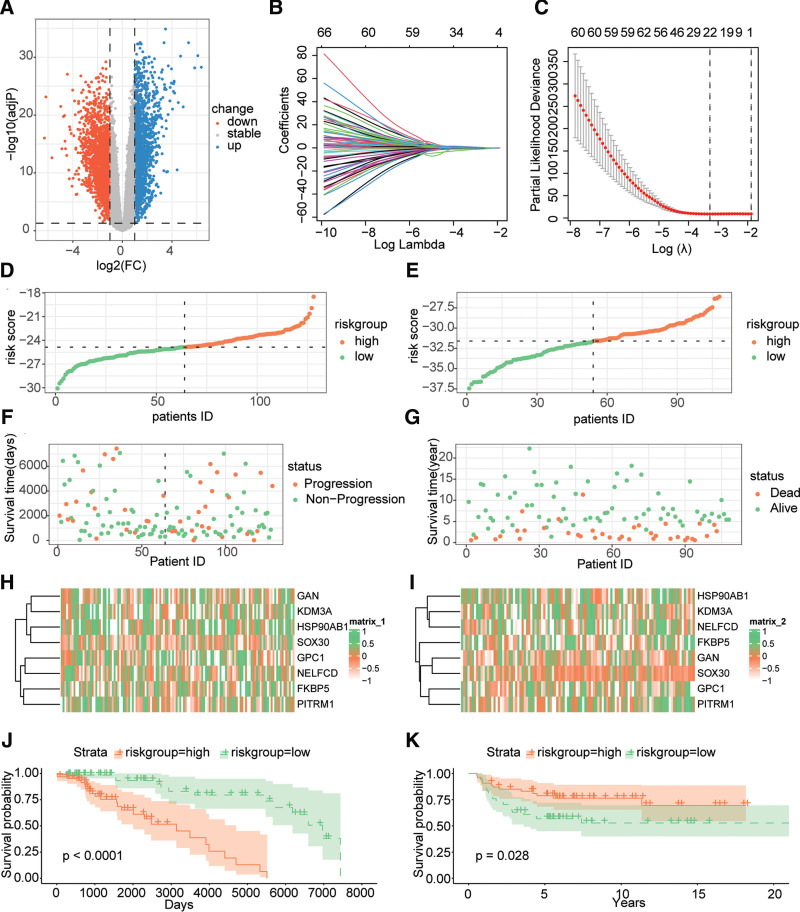
Development of TGCT prognostic risk features derived from MMRG. (A) Volcano plot of differentially expressed genes between cluster 1 and cluster 2. (B–C) Lasso Cox regression analysis based on prognostic-associated differentially expressed genes. (D–E) Stratification of high-risk and low-risk groups in the training and validation cohorts. (F–G) Scatter plots of patient survival time and outcome in the training and validation cohorts. (H–I) Heatmaps of the expression of model-incorporated genes in the training and validation cohorts. (J–K) Kaplan–Meier survival curve analysis for patients in high-risk and low-risk groups in the TCGA and GEO cohorts, respectively. TCGA = the cancer genome atlas, TGCT = testicular germ cell tumor.

The differential expression analysis revealed (Fig. 6A) that there were 3636 genes that showed significant differential expression (adjusted *P* value < .05, |logFC|>1) between cluster1 and cluster2. To establish prognostic risk features for TGCT, we selected differentially expressed genes with *P* < .01 in both clusters and identified 185 genes significantly associated with TGCT prognosis. Lasso Cox regression analysis further reduced the prognostic gene set to 21 (Fig. 6B and C), of which 8 were deemed independent prognostic factors for TGCT and used to develop a risk score model: risk score = −1.2777996 * FKBP5 + 1.7058897 * GAN −0.7473239 * GPC1 −1.2051032 * HSP90AB1 −1.3910335 * KDM3A −2.7566646 * NELFCD + 2.0835098 * PITRM1 + 1.5523928 * SOX30. The TCGA-TGCT and GEO cohorts were stratified into high-risk and low-risk groups based on this model (Fig. 6D and E), and the survival time, outcome, and expression of these prognostic genes in patients are presented in Figure 6F–I. Survival analysis demonstrated that patients in the high-risk group had significantly worse prognosis than those in the low-risk group in both TCGA-TGCT cohort (Fig. 6J, log-rank test: *P* < .0001) and GEO cohort (Fig. 6K, log-rank test: *P* = .028).

### 3.7. Establishment and evaluation of nomogram for prognostic evaluation of TGCT patients

**Figure 7.**
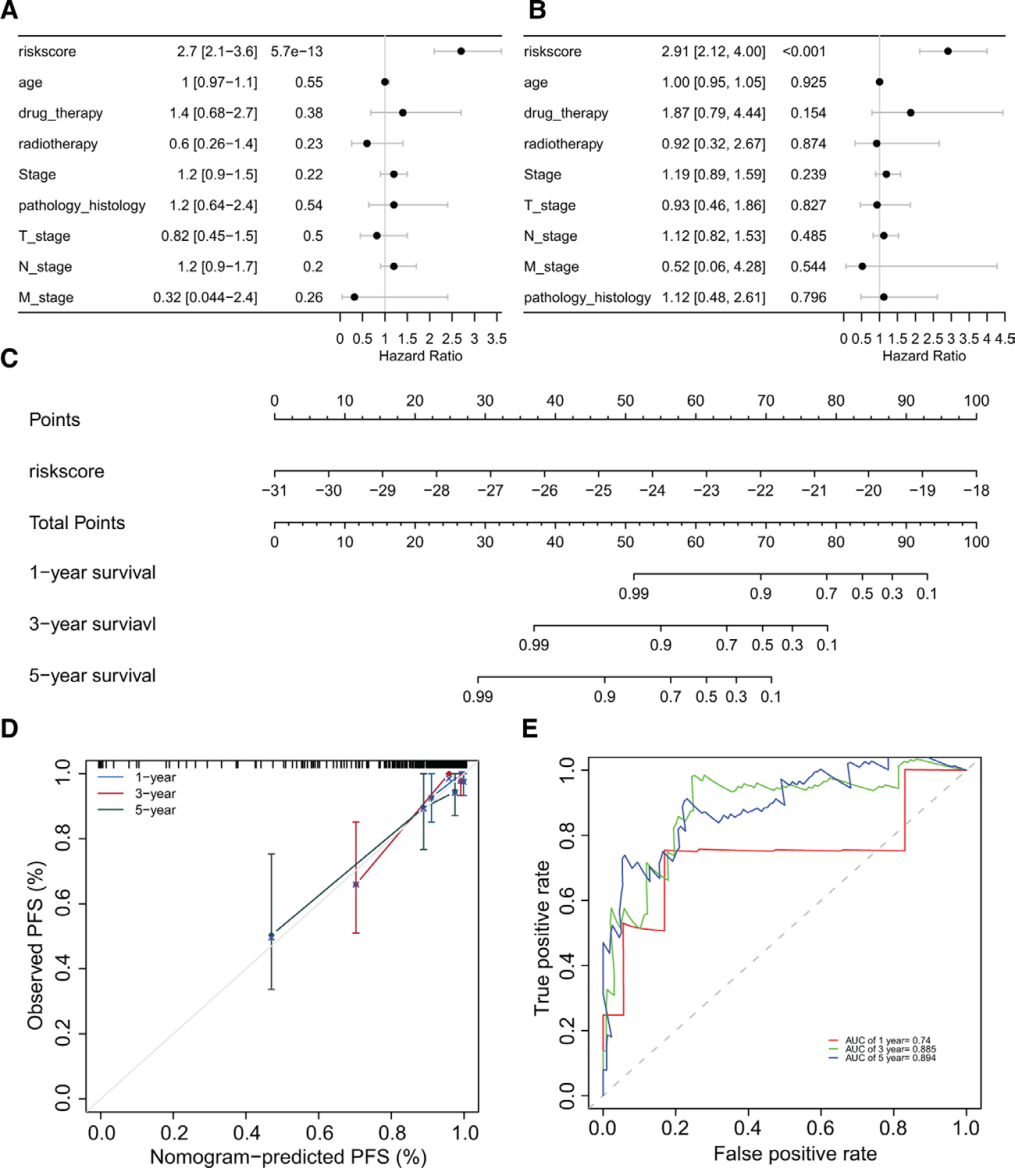
The establishment and evaluation of nomogram: (A–B) univariate and multivariate Cox regression analyses of clinical-pathological features and risk score in TCGA-TGCT cohort, (C) nomogram composed by independent prognostic risk score derived from MMRG, (D) calibration curve analysis evaluating the prediction accuracy of nomogram for predicting PFS at various time points in TGCT patients, and (E) receiver operating characteristic curve analysis evaluating prediction accuracy of nomogram for predicting PFS at different time points in TGCT patients with AUC values shown. PFS = progression-free survival, TCGA = the cancer genome atlas, TGCT = testicular germ cell tumor.

In order to evaluate the prognosis of TGCT patients, we performed univariate and multivariate Cox regression analyses on risk score and clinical-pathological features derived from TCGA-TGCT cohort. The results indicated that only the risk score derived from MMRG was identified as an independent prognostic factor for TGCT (Fig. 7A and B). Based on this, we constructed a nomogram using the MMRG-derived risk score to predict the 1-, 3-, and 5-year PFS of TGCT patients (Fig. 7C). The calibration curves demonstrated that the predicted PFS by this nomogram was in good agreement with the actual outcomes (Fig. 7D). Furthermore, receiver operating characteristic curve analysis showed that our nomogram had high predictive accuracy with AUC values of 0.74, 0.885, and 0.894 for predicting PFS at 1-, 3-, and 5-years respectively (Fig. 7E).

## 4. Discussion

In this study, we have identified fifteen MMRGs that are significantly associated with progression-free survival in patients with TGCT. These genes have been widely implicated in the initiation and progression of cancer. For example, Nudix hydrolase 1 is aberrantly overexpressed in various human malignancies and is a commonly recognized poor prognostic indicator for multiple cancers such as colorectal cancer,^[18]^ clear cell renal cell carcinoma,^[19]^ and hepatocellular carcinoma.^[20]^ Phosphatase, orphan 1, serves as a key metabolic biomarker associated with diffuse large B-cell lymphoma.^[21]^ Serine palmitoyltransferase small subunit A is a neuroglial tumor prognostic marker that correlates with tumor-infiltrating immune cells and oxidative stress.^[22]^ HDAC3, an epigenetic drug target, is currently being regarded as a potential therapeutic strategy against various cancers.^[23,24]^ Research has shown that deoxythymidylate kinase plays a role in cancer progression and chemotherapy response and may serve as a potential prognostic factor for multiple cancers.^[25,26]^ Mediator complex subunit 10 is an important kinase module that drives the tumorigenicity and refractory phenotype of bladder urothelial carcinoma by upregulating hsa-miR-590.^[27]^ Although these genes have been extensively studied for their roles in various cancers, their involvement in TGCT tumorigenesis and progression remains unclear.

Based on the prognostic-associated MMRG expression characteristics, we identified 2 molecular subtypes of TGCT which exhibit significant heterogeneity in clinical-pathological features, prognosis, immune infiltration, somatic mutations, and drug sensitivity. These subtypes have potential clinical value in future personalized treatment plan design. NK cells can recognize and kill cancer cells, infectious pathogens, and damaged cells. They also secrete multiple cytokines to modulate immune responses. Therefore, the better prognostic feature of Cluster1 may originate from its higher level of NK cell infiltration. In addition, we elucidated the significant correlation between prognostic-associated MMRGs and tumor immune cell infiltration, suggesting their potential roles in shaping the TGCT tumor immune microenvironment. The function of immune cells depends on specific mitochondrial metabolic programs that include nutrient oxidation, macromolecule synthesis and posttranslational modifications.^[28]^ Studies have shown that HDAC3 promotes immune escape in breast cancer cells by downregulating microRNA-130a-3p and selectively inhibits lymphoma immune surveillance by activating HDAC3.^[29,30]^ However, most mechanisms involved in gene regulation for immune modulation remain unclear and require further experimental exploration.

Through gene set enrichment analysis, we have identified that immune-related biological processes were significantly enriched, while extracellular matrix (ECM)-related biological processes were significantly underrepresented in the subtypes with favorable prognosis. The alterations in ECM density and composition within tumors are known to play a critical role in promoting tumor growth and progression by influencing stiffness and degradation. Cancer-associated fibroblasts represent the primary drivers of ECM stiffening and degradation. Notably, the significant differences observed in CAF scores between TGCT molecular subtypes derived from MMRG may serve as an important driving factor for ECM changes.^[31]^

Furthermore, we found that the PI3K-AKT signaling pathway was significantly suppressed in the subtypes with better prognosis. This pathway is known to play a crucial role in various cellular processes and is aberrantly activated in cancer, thereby contributing to tumorigenesis and development. Research has demonstrated that PI3K/Akt signaling pathway cascade inhibitors are among the most effective therapeutic strategies for treating cancer, whether used alone or in combination with other therapies.^[32]^ Therefore, our findings suggest that targeting the PI3K-AKT signaling pathway and regulating ECM components and density could be a promising approach for effectively treating TGCT subtypes with better prognosis.

Finally, we developed a risk signature using differentially expressed genes between subtypes for predicting the PFS of TGCT patients. The risk signature, which comprises 8 genes, demonstrated outstanding prognostic performance in predicting PFS. Furthermore, the risk signature was identified as an independent prognostic factor for PFS in TGCT patients and was utilized to construct a nomogram for predicting 1-, 3-, and 5-year PFS. However, it is important to acknowledge that these subtypes and risk signature were derived from publicly available databases, thus necessitating further validation using clinical samples. Additionally, the functional roles and underlying mechanisms of most genes in TGCT remain largely unexplored and warrant further experimental investigation.

## 5. Conclusion

In summary, our study has provided insights into the prognostic relevance of 15 MMRGs and identified 2 molecular subtypes in TGCT that demonstrate significant differences in prognosis, immune infiltration, and treatment response based on these genes. Additionally, we have developed a highly effective TGCT prognostic risk signature. These subtypes and risk signature hold potential for personalized treatment strategies for TGCT patients in future clinical applications and require further validation.

## Author contributions

**Conceptualization:** Dandan Qiu, Lingling Gao.

**Formal analysis:** Dandan Qiu, Lingling Gao, Shuo Zhang.

**Funding acquisition:** Xingwei Yu.

**Methodology:** Dandan Qiu, Lingling Gao.

**Project administration:** Dandan Qiu, Lingling Gao, Shuo Zhang, Gang Lin.

**Resources:** Shuo Zhang.

**Supervision:** Xingwei Yu.

**Writing – original draft:** Dandan Qiu, Gang Lin, Xingwei Yu.

**Writing – review & editing:** Xingwei Yu.
